# Application of a Broad Range Lytic Phage LPST94 for Biological Control of *Salmonella* in Foods

**DOI:** 10.3390/microorganisms8020247

**Published:** 2020-02-13

**Authors:** Md. Sharifull Islam, Yang Zhou, Lu Liang, Ishatur Nime, Ting Yan, Stephan P. Willias, Md. Zakaria Mia, Weicheng Bei, Ian F. Connerton, Vincent A. Fischetti, Jinquan Li

**Affiliations:** 1 Key Laboratory of Environment Correlative Dietology, College of Food Science and Technology, Huazhong Agricultural University, Wuhan 430070, China; smbgb101287@yahoo.com (M.S.I.); smbgb101287@gmail.com (I.N.); yantingau@163.com (T.Y.); 2 College of Veterinary Medicine, Huazhong Agricultural University, Wuhan 430070, China; beiwc@mail.hzau.edu.cn; 3 State Key Laboratory of Agricultural Microbiology, Huazhong Agricultural University, Wuhan 430070, China; zhouyang@mail.hzau.edu.cn; 4 College of Fisheries, Huazhong Agricultural University, Wuhan 430070, China; 5 Division of Microbiology, Brewing & Biotechnology, University of Nottingham, Sutton Bonington Campus, Loughborough, Leicestershire LE12 5RD, UK; sbzll3@nottingham.ac.uk (L.L.); scziac@exmail.nottingham.ac.uk (I.F.C.); 6 Department of Infectious Diseases and Immunology, University of Florida, Gainesville, FL 32611-2015, USA; swillias@ehs.ufl.edu; 7 Department of Microbiology, Jagannath University, Dhaka 1100, Bangladesh; mmzakaria@yahoo.com; 8 Laboratory of Bacterial Pathogenesis and Immunology, The Rockefeller University, New York, NY 10065-6399, USA; vaf@mail.rockefeller.edu

**Keywords:** phage, LPST94, *Salmonella*, characterization, biological control

## Abstract

*Salmonella*, one of the most common food-borne pathogens, is a significant public health and economic burden worldwide. Lytic phages are viable alternatives to conventional technologies for pathogen biocontrol in food products. In this study, 40 *Salmonella* phages were isolated from environmentally sourced water samples. We characterized the lytic range against *Salmonella* and among all isolates, phage LPST94 showed the broadest lytic spectrum and the highest lytic activity. Electron microscopy and genome sequencing indicated that LPST94 belongs to the *Ackermannviridae* family. Further studies showed this phage is robust, tolerating a wide range of pH (4–12) and temperature (30–60 °C) over 60 min. The efficacy of phage LPST94 as a biological control agent was evaluated in various food products (milk, apple juice, chicken breast, and lettuce) inoculated with non-typhoidal *Salmonella* species at different temperatures. Interestingly, the anti-*Salmonella* efficacy of phage LPST94 was greater at 4 °C than 25 °C, although the efficacy varied between different food models. Adding phage LPST94 to *Salmonella* inoculated milk decreased the *Salmonella* count by 3 log_10_ CFU/mL at 4 °C and 0.84 to 2.56 log_10_ CFU/mL at 25 °C using an MOI of 1000 and 10000, respectively. In apple juice, chicken breast, and lettuce, the *Salmonella* count was decreased by 3 log_10_ CFU/mL at both 4 °C and 25 °C after applying phage LPST94 at an MOI of 1000 and 10,000, within a timescale of 48 h. The findings demonstrated that phage LPST94 is a promising candidate for biological control agents against pathogenic *Salmonella* and has the potential to be applied across different food matrices.

## 1. Introduction

The diarrheal disease salmonellosis caused by non-typhoidal *Salmonella* spp. is one of the most frequent foodborne illnesses worldwide [1]. The major symptoms of *Salmonella* infection are abdominal pain, vomiting, inflammatory diarrhea, fever, and headache [2,3]. In the United States alone, *Salmonella* is annually responsible for 11% of illnesses, 35% of total hospitalizations, and 28% of deaths associated with food-borne diseases [4]. An outbreak was recently reported in Iowa, USA, where over 250 people were ill from consuming *Salmonella* contaminated chicken salad. Among them, one person died and more than 90 others were hospitalized [5]. In China, non-typhoidal *Salmonella* spp. are of concern with an estimated 414.8 cases occurring annually in Guangdong Province [6]. *Salmonella* commonly contaminates an extensive range of food products, including meat, eggs, milk, vegetables, and fruit juices. *Salmonella* contamination in food preparations typically arise from the natural association of the bacteria with products of animal origin and cross-contamination during processing services [7,8].

Conventional control measures such as heat treatment or chemical preservatives can control pathogens, including various *Salmonella* serovars, in food products [9,10,11]. However, these measures run the risk of negatively affecting the taste of food products and reducing also nutritional availability [12,13]. Many chemical preservatives have been reported to have the potential for adverse side effects, including allergic contact dermatitis, convulsion, hives, asthma, diarrhea, intestinal hemorrhage etc. [14,15]. Thermal processing can negatively influence the quality of food products by destroying vitamins thus reducing their nutritional value [13]. Moreover, advanced glycation end-products (AGEs) that are produced by heat treatment have been considered as health-threatening complications [16]. Current methods to treat acute *Salmonella* gastroenteritis are dependent upon supportive care and antibiotic regimens. The overzealous application of antibiotics, especially in the farm setting, has led to the selection of antimicrobial-resistant strains [17,18], which have contaminated food and water systems, and pose a worldwide threat. Under these circumstances, the measures employed to control *Salmonella* must not lead antimicrobial resistance, which has garnered interest in biological controls.

Although using phages for therapeutic purposes or as biocontrol agents is not entirely a new idea, in recent years, urged by the increasing development of drug-resistant bacteria, interest in phage therapy has been rekindled [19,20]. Phage as therapy offers many attractive advantages including pathogen-targeting, host-specificity, rapid killing, and self-replicating potential [21,22]. Phages are obligate viral parasites of bacteria which, through interactions with unique bacterial surface receptors, infect specific prokaryotes bearing the distinct molecular signatures. As such, phages exhibit extreme host-specificity which enables the selective targeting of certain bacterial genera or species. Considering phages only infect bacteria, they elicit little harm to eukaryotic cells, thereby affording a high degree of safety [23,24].

Phages have been used for inactivation and control of food-borne pathogens, such as *Salmonella* in diverse food matrices [25,26,27,28,29,30]. Also, commercially available phage products such as SalmoFresh, Armament, Salmonelex have been used for inactivation and control *Salmonella* in food products [30,31]. Since bacteria and phage have co-evolved for billions of years, bacteria have developed multiple defense systems against phage [32,33]. It requires us to continuously provide new promising phages with relatively broad lytic range and high lytic activity for practical application. In the present study, 40 environmentally acquired phages were isolated and screened to determine their bacterial lytic range and lytic activity. The newly isolated phage LPST94 showed the greatest potential in respect of control zoonotic *Salmonella* in diverse food matrices.

## 2. Materials and Methods

### 2.1. Bacterial Strains and Culture Conditions

*Salmonella enterica* (UK-1, ATCC 13311) was used as the host strain for phages isolation. A total of 65 different bacterial strains, consisting of 40 *Salmonella* strains encompassing 11 distinct serovars as well as a cohort of 25 non-*Salmonella* strains, were used to determine the phage lytic range (Table S1). All bacterial strains were cultured by the streak plate method on tryptic soy agar (TSA; Difco^TM^, BD, USA) followed by overnight incubation at 37 °C.

### 2.2. Enrichment, Isolation, Purification, and Preparation of Phages

A total of 40 putative different phages were isolated from the environmentally sourced water samples collected in Wuhan, China in accordance with previously described methods [34,35]. Enrichment for phage isolation was modified from previously published methods [36]. In brief, 10 mL of exponential growth phase *Salmonella* cultures were mixed with 40 mL 2-YT broth medium (1.6 g of peptone, 1.0 g of yeast extract, and 0.5 g NaCl, in 100 mL of distilled water; pH 7.4) and 10 mL of filtered water sample at a ratio of 1:4:1 (v/v/v) to amplify the collected phages. Amplified phages were isolated by centrifugation at 8000× *g* for 15 min and filtration using 0.22 μm pore size disposable sterile syringe filters (Millipore, Ireland). The double layer agar method was used to determine the titer of the phage stock. Dilutions of the phage stock (100 μL each) were made in sterile SM buffer (10 mM NaCl, 10 mM MgSO_4_, 50 mM Tris:HCl, pH 7.5), mixed with a suspension of exponential phase *Salmonella* (about 10^9^ CFU/mL, 100 μL) and added to 4 mL of molten (45 °C ≤ temperature ≤ 50 °C) tryptone soya broth (TSB) with agar (0.7%). The mixtures were then poured onto the surface of tryptic soy agar (TSA) plates and were allowed to set at room temperature for 5 min. Thereafter, the plates were incubated at 37 °C for 24 h, and resulting plaques were quantified. To purify the phages, individual plaques were picked using a pipette or a wire loop, and then suspended in TSB with exponential phase *Salmonella* at 37 °C for 24 h. The suspension was centrifuged (8000× *g* for 15 min) and filtered again using 0.22 μm filters used as a single phage culture. The purification process was repeated at least four times, and then confirmed pure individual phage stock. High titer stocks of phage were prepared by mixing 100 μL of the phage stock (about 9 log_10_ PFU/mL) with 100 μL culture of *Salmonella* in 50 mL of TSB broth incubated for 12 h at 37 °C. The culture was centrifuged at 10,000× *g* for 10 min and the supernatant was filtered through a disposable sterile syringe filter (0.22 μm pore Millipore, Ireland). The purified phages were stored at 4 °C.

### 2.3. Screening of Phages Based on Spot Test and Lytic Capacity

#### 2.3.1. Lytic Spectrum Determination by Spot Test

The ability of phages to lyse different serovars of bacteria was determined by spot test. In brief, 5 μL of phage lysates were spotted onto lawns formed by the aforementioned 65 bacterial strains on TSA plates. The plates were incubated at 37 °C for 24 h. After incubation, any bacterial lawn with formation of clear plaques were considered as phage sensitive.

#### 2.3.2. Lytic Activity of Phage LPST94

Lytic activity experiments were performed according to previously described method [35,37]. In brief, 100 μL fresh overnight cultures of *Salmonella* (7 log_10_ CFU/mL) were added to 100 μL of phage suspensions (MOIs 100, 10, 1, and 0.1) in a 96 well plate. The control group consisted of plain TSB medium in place of phage LPST94. Optical density (OD_600_) was measured with a microplate reader (Infinite M200 Pro, Tecan, 140 Switzerland) at 37 °C, with a reading recorded every 1 h for a total duration of 24 h. Response curves of lytic activity were generated using PRISM software.

### 2.4. Efficiency of Plating

Efficiency of plating (EOP) was determined as described previously [35,38]. Test bacterial strains were grown overnight at 37 °C and 100 μL of each of the test bacterial cultures was used in double layer assays together with 100 μL of 10^6^–10^9^-fold diluted phage lysate. The plates were incubated overnight at 37 °C. After incubation, the number of plaques were counted and the relative EOP was subsequently calculated (average PFU on test bacteria/average PFU on host bacteria). The average EOP values were classified as follows: +++, EOP 0.5 to 1.0; ++, EOP 0.2 to <0.5; +, 0.001 to <0.2; -, (<0.001).

### 2.5. Morphological Observation of Phage LPST94

Prior to transmission electron microscopy, high titer phage LPST94 was obtained by pelleting phage with ultracentrifugation at 40,000× *g* for 1 h, followed by re-suspending pellet in small volumes. Phage LPST94 was diluted into phosphate-buffered saline (PBS) and was negatively stained with 0.5% phosphotungstic acid (PTA) [34,39]. Thereafter, the phage was fixed on a copper grid and the images of the phages were captured using a Philips CM12 transmission electron microscope (Hitachi H-7000FA, Tokyo, Japan), at Wuhan Institute of Virology (China Academy of Sciences, Wuhan, China) and analyzed via Digital Micrograph Demo 3.9.1 software.

### 2.6. Genomic Analysis of Phage LPST94

Genomic DNA of the phage LPST94 was extracted and purified as described previously [40]. The genome was sequenced on the HiSeq platform (Illumina, San Diego, CA, USA) by means of a paired-end library with a 150 bp read length. The sequences were assembled by using Newbler (v3.0) resulting in a unique contig. Putative coding DNA sequences (CDSs) were identified by Glimmer 3.0 [41]. Functional annotations of CDSs were identified by searching against the nr protein database using BLASTP [42]. The database ARG-ANNOT (http://backup.mediterranee-infection.com/article.php?laref=282&titre=arg-annot) was used to detect the antimicrobial-resistant genes (ARGs) in phage. The database VFDB (http://www.mgc.ac.cn/VFs/main.htm) was used to detect the virulent factor in phage. The genome map constructed by using BRIG.jar software. The complete genome sequence of phage LPST94 has been deposited in GenBank under the accession number MH523359.

### 2.7. One-Step Growth Curve

One-step growth curve experiments were carried out to determine the phage LPST94 latent period and burst size essentially as described previously [43,44]. Briefly, *S.* Typhimurium UK-1 was grown to mid-log phase and 1 mL of bacterial culture (about 7.3 log_10_ CFU/mL) was combined to 1 mL of phage lysate (about 4.3 log_10_ PFU/mL) to achieve a multiplicity of infection (MOI) of 0.001. The mixture was incubated for 20 min at 37 °C and was subsequently centrifuged at 7000× *g* for 2 min. The supernatant was discarded, and the pellet was washed twice with TSB broth. The pellet was then suspended in 10 mL of TSB broth and incubated at 37 °C, and 200 μL samples collected at 10 min intervals over 3 h from which phage titers were determined by the double layer agar plate method [45] to establish a one-step curve. The latent period was defined as the time interval between absorption and the beginning of the first burst. The burst size was calculated as the ratio of the final number of phage particles to the initial number of infected host cells at the beginning of the test [33,34].

### 2.8. pH and Thermal Tolerance of the Phage LPST94

To determine the effect of pH on the stability of phage LPST94, phage lysates (8.49 log_10_ PFU/mL) were added to tubes containing sterile buffered peptone water (BPW) with pH values ranging from 2 to 13 adjusted with NaOH or HCl. The tubes were allowed to incubate at 37 °C for 60 min. Thereafter, the phage solutions were serially diluted and the recovered phage titers were determined using *S.* Typhimurium UK-1 as a host by means of the double-layer agar method [46]. For the evaluation of thermal stability, the phage LPST94 lysates (8.49 log_10_ PFU/mL) were incubated at 30 °C, 40 °C, 50 °C, 60 °C, 70 °C, and 80 °C for 1 h [37]. After 30 or 60 min of incubation, aliquots were extracted to determine phage titers through the application of the double-layer agar method.

### 2.9. Phage Stability in Diverse Food Samples

Phage stability experiments were conducted according to a previously described method [47] with little modification. Briefly, the phage was added in the milk, apple juice, chicken breast, and lettuce to reach a final titer of 7 log_10_ PFU/mL. All inoculated samples (milk, apple juice, chicken breast, and lettuce) were incubated at 25 °C for 0, 1, 3, 6, 12, 24, and 48 h. At each time-point, food samples (milk or apple juice or pre-cut chicken breast or lettuce) were taken to measure phage titer by using double-layer agar method.

### 2.10. Ability of Lysogenic Formation of the Phage

To confirm that phage LPST94 was unable to form lysogen, lysogenic induction was performed with phage-resistant bacterial colonies [48]. A single colony of purified bacteria was inoculated into tube with 5 mL TSB medium and stress-induced with mitomycin C (Sigma Chemical, St. Louis, MO, USA) to a final concentration of 1 μg/mL. The samples were incubated overnight at 37 °C. After incubation, the bacteria were pelleted by centrifugation at 10,000× *g* for 10 min and the supernatant was examined for the presence of phages. The presence of a clear zone after stress-inducing indicates that bacteria contain prophage in their genome.

### 2.11. Biological Control of Salmonella in Foods Using Phage LPST94

#### 2.11.1. Testing in Milk and Apple Juice

Pasteurized milk and apple juice were purchased from a local supermarket. *Salmonella* biocontrol experiments using phage LPST94 were conducted at 4 °C (refrigeration temperature) and 25 °C (room temperature) described previously [49]. Experimental groups were temperature acclimated for 20 min before 9 mL of milk or apple juice was spiked with *S.* Typhimurium ATCC 14028 or by a mixture of *S.* Typhimurium ATCC 14028 and *S.* Enteritidis ATCC 13076 to a final count of 3.5 log_10_ CFU/mL. Phage LPST94 was then added to an MOI of 1000 or 10,000. Aliquots were removed after 0, 1, 3, 6, 12, 24 and 48 h incubation. For *Salmonella* enumeration, 100 μL of 10-fold serial diluted samples were plated, giving the detection limit of 1 CFU/100 μL, and the plates were incubated for 48 h at 37 °C.

#### 2.11.2. Testing in Chicken Breast and Lettuce

Chicken breast and lettuce were obtained from a local supermarket then sliced aseptically in the laboratory. The chicken breast was cut into pieces (1 cm × 1 cm square and 0.5 cm thick) using a sterile scalpel blade on the sterile station board. Similarly, the inner leaves of the lettuce were cut into pieces (1 cm × 1 cm square) using a sterile sharp knife. The 1 cm^2^ food sections were placed in the center of the sterile petri-dishes and inoculated *S.* Typhimurium ATCC 14028 or a mixture of *S.* Typhimurium ATCC 14028 and *S.* Enteritidis ATCC 13076 to a final viable count of 3 log_10_ CFU/cm^2^. Phage LPST94 was then transferred on the sample surface with an MOI of 1000 or 10,000. These petri-dishes were incubated at 4 °C or 25 °C for 48 h with lid on. After incubation, the sample was transferred to 2 mL Eppendorf tube and 1 mL PBS buffer was added to the sample in a sterile environment. The chicken breast and lettuce samples were homogenized with sterile bars and vortexed. The proportions of recoverable bacteria among the control group and the experimental group were determined by direct spread plate methods [47].

### 2.12. Statistical Analysis

Bacteria and phage counts were determined by duplicate plating, and all experiments were completed using a minimum of three biological replicates. Results are presented as mean value and variance were determined by the standard deviation from the mean. Statistical analyses were performed with PRISM software. Multivariate comparisons were performed using nonparametric one-way analysis of variance (ANOVA) with Bonferroni’s multiple-comparison posttest.

## 3. Results

### 3.1. Isolation and Screening of Phage

A total of 40 putative different phages were isolated from environmentally-sourced water samples with either *Salmonella enterica* serovar Typhimurium UK-1 or ATCC 13311 as host bacteria. Differences in plaque size and turbidity were observed between each isolate. A collection of *Salmonella* strains was applied to determine the host range of all phage isolates with the same titer. The results of spot test showed that, of these phages, 22.5% of the isolates (9 out of 40) formed clear plaques and were capable of lysing more than 50% of the *Salmonella* test strains (Table 1), whereas the rest were specific to the initial isolation host. Spot tests indicated that phage LPST94 had the broadest lytic range. Phage LPST94 lysed all 40 *Salmonella* strains tested in this study that represent 11 *Salmonella* serovars and include drug-resistant *Salmonella*. However, all the phage isolates were unable to lyse *E. coli* or any of the Gram-positive and Gram-negative bacteria tested (Table 1).

**Figure 1:**
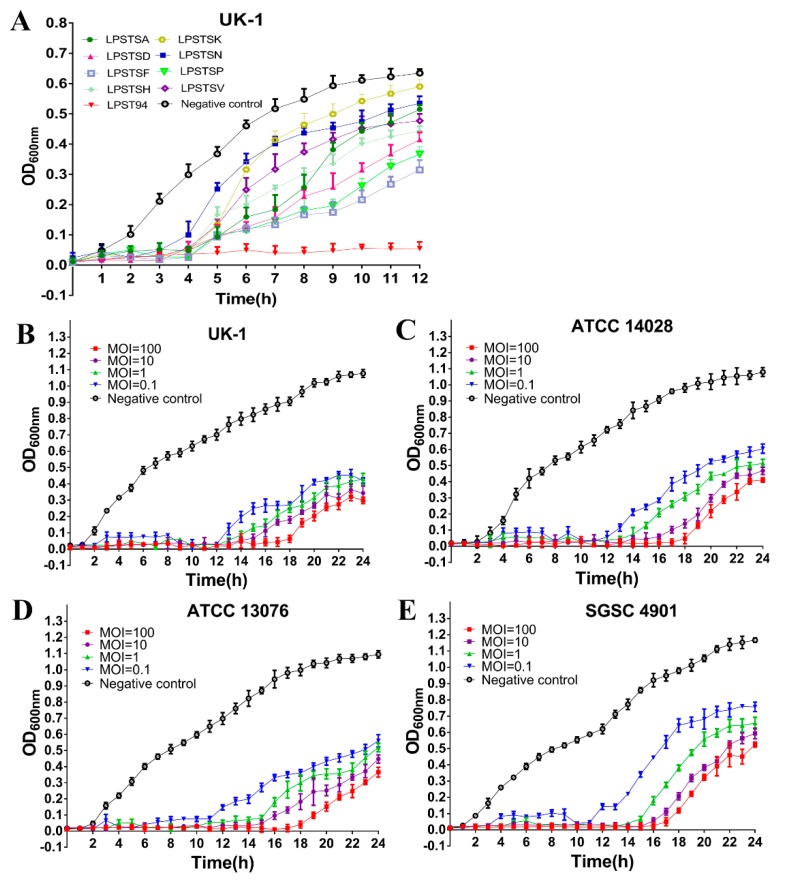
(**A**) Comparison of the lytic ability of selected phages using *S. enterica* serovar Typhimurium (UK-1) as a host at multiplicity of infection (MOI) of 1 in tryptone soy broth; Lytic ability of phage LPST94 to lyse *S.* Typhimurium and *S.* Enteritidis in tryptone soy broth medium at MOIs of 100, 10, 1, and 0.1 at 37 °C in vitro. (**B**) *S.* Typhimurium UK-1, (**C**) *S.* Typhimurium ATCC 14028, (**D**) *S.* Enteritidis ATCC 13076, and (**E**) *S.* Enteritidis SGSC 4901. Values represent means with the standard deviation of three replicates of each time point.

The lytic activity of these 9 wider lytic range phages were further evaluated using a challenge test against their initial isolation host *Salmonella enterica* serovar Typhimurium UK-1. As shown in Figure 1A, LPST94 successfully inhibited the growth of host bacteria over 12 h whereas the growth of host bacteria recommenced 4 h (*p* < 0.05) after infection with all other phages (LPSTSA, LPSTSD, LPSTSF, LPSTSH, LPSTSK, LPSTSN, LPSTSP, and LPSTSV). To further establish the inhibitory activity of LPST94, we examined two *S.* Typhimurium (Figure 1B,C) and two *S.* Enteritidis (Figure 1D,E) strains for 24 h over a range of MOI (0.1, 1, 10 and 100). The growth of both *S.* Typhimurium and *S.* Enteritidis were inhibited for 11–12 h (*p* < 0.05) after adding phage LPST94 at MOIs of 0.1, 1, 10 and 100. Although the bacteria started growing after 12 h, a significant differences were observed when applying different MOIs (*p* < 0.05; paired ANOVA). Generally, a greater sustained inhibitory effects were observed when phage was applied at higher MOIs.

### 3.2. Relative Replication Efficiency of Phage LPST94

As noted by Mirzaei et al. single dilution spot tests may show false positive results by lysis from without, bacteriocins in the phage lysate, or the existence of prophages [50]. Here, an EOP test was carried out to confirm the host range of phage LPST94 (Table 2). This phage had a high efficiency (0.5 to 1.0) to infect the majority of *S*. Typhimurium strains compared with its isolation host *S.* Typhimurium UK-1, but the EOP values were moderate (0.2 to <0.5) for some *S.* Enteritidis and drug-resistant *Salmonella* strains. The EOP of LPST94 phage was also analyzed on 18 drug-resistance *Salmonella* strains from our collection (the drug-resistance profiles of the 18 isolates of *Salmonella* are presented in Table S4). Clear plaques were observed for all 18 strains with relative EOP values ranging from 0.001 to <0.2. These results suggest that the phage LPST94 is capable of lysis over a range of *Salmonella* that contaminate foods (Table 2). 

### 3.3. Phage Morphology and Genomic Analysis

**Figure 2:**
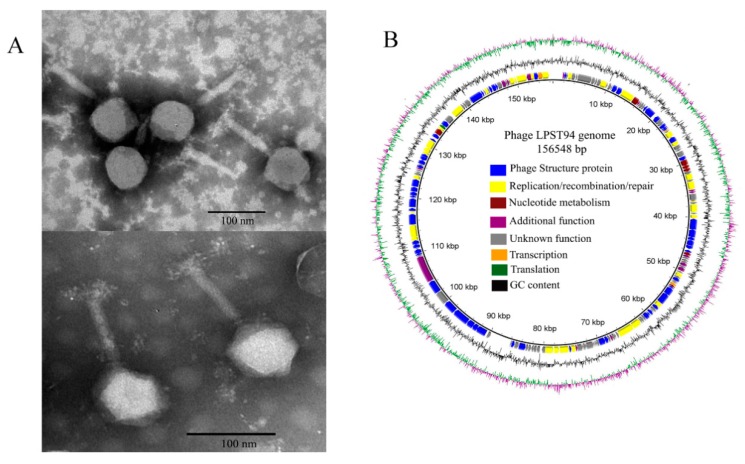
Morphological and genomic characteristics of phage LPST94. (**A**) TEM image of phage LPST94; Bar 100 nm, and (**B**) Genome map of phage LPST94. Patterns were divided into four circles: the full length of the genome was indicated in the first circle; the open reading frame was indicated in the second circle, and the clockwise arrow and the counterclockwise arrow represented the forward reading frame and the reverse reading frame, respectively; GC content was indicated in the third circle; while on the fourth circle, GC skew of G-C/G+C was indicated as green and purple, and green meant the values of GC skew greater than 0 and purple meant the values less than 0. The open reading frames marked with the color of each gene refers to the functional category: phage structure (blue), replication/recombination/repair (yellow), nucleotide metabolism (maroon), transcription (orange), translation (green) additional function (purple) and hypothetical proteins (grey). The genome map generated by the BRIG.jar software.

Transmission electron microscopic examination of phage LPST94 showed that it has an icosahedral head and a long, rigid and relatively thick contractile tail that terminates in a baseplate with spikes. Its head is 67.60 ± 2.30 nm in diameter (*n* = 6) and its tail is 116.30 ± 4.10 nm long (*n* = 6) (Figure 2A). The morphology suggests that LPST94 belongs to the *Ackermannviridae* or *Myoviridae* family, in the order of *Caudovirales*. The genome size of LPST94 was 156,548 bp with a GC content of 44.6%. BlastN analysis shows the genome of phage LPST94 has a high degree of homology with phage PhiSH19 in the database (Genbank Acc. No. JN126049). Compared with PhiSH19 phage, LPST94 has additional ORFs encoding tail fiber and tail spike proteins. The multiple tail fiber and tail spike proteins might be the reason for the broad lytic range observed. The genome of phage LPST94 contained 197 predicted ORFs (Figure 2B and Table S2) responsible for structure, replication/recombination/repair, nucleotide metabolism, transcription, translation and additional phage functions. They also include structural genes for phage assembly including the major capsid protein, prohead core protein, prohead protease, head completion protein, and tail/neck structure proteins. The genome encodes all the components necessary to complete the phage structure. The replication/recombination/repair gene modules encode replication proteins (DNA helicases, DNA polymerases, rIIA and rIIB proteins, DNA primase etc.) and recombination/repair proteins (single-stranded DNA binding protein, RecA-like recombination protein, DNA repair/recombination protein, recombination protein subunit), suggesting that this phage has its own replication/recombination/repair system. The module for nucleotide metabolism encodes putative dUTP diphosphatase, thymidylate synthase, ribonucleotide diphosphate reductase beta subunits etc. Gene functions fortranscription/translation are encoded by a sigma transcription factor, Gp33 late promoter transcription factor and RegA translational repressor protein. The LPST94 genome encodes a number of additional proteins, such as serine/threonine phosphatase for amino acid biosynthesis, PhoH-like protein for phosphate starvation and glutaredoxin (Figure 2B and Table S2). ARG-ANNOT database, VFDB database and BLASTP searches indicate that the LPST94 genome does not encode recognizable virulence factors, toxins, drug-resistant or integrase encoding genes, suggesting that the phage is safe and has the potential to be developed as an alternative antimicrobial agent. BLAST analysis confirmed that LPST94 belongs to the *Ackermannviridae* family, in the order of *Caudovirales* (Table S3). The complete genome sequence of phage LPST94 was deposited in GenBank under the accession number MH523359.

### 3.4. Characteristics of Phage LPST94

**Figure 3:**
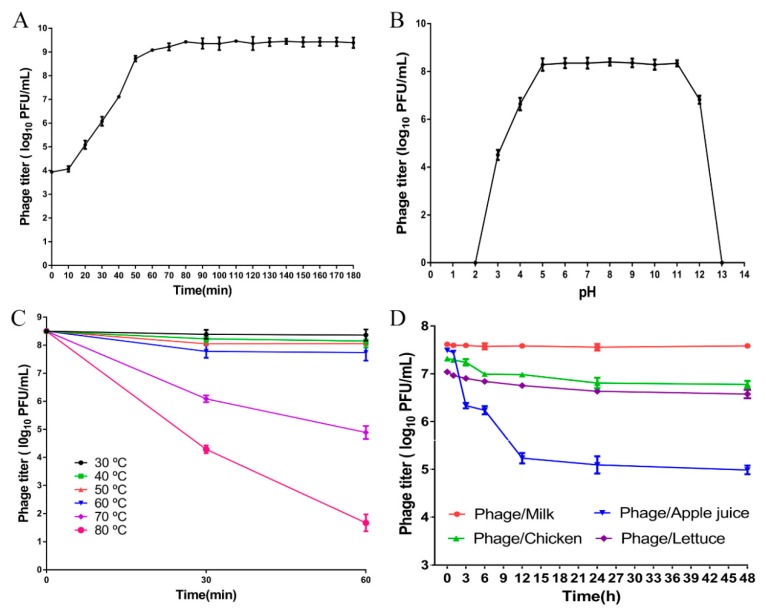
Characteristics of phage LPST94. (**A**) One-step growth curves of phage LPST94 with *Salmonella enterica* serovar Typhimurium UK-1 host infected at 37 °C, (**B**) pH tolerance of phage LPST94 (pH 2 to 13), (**C**) Temperature tolerance of phage LPST94 (30 °C to 80 °C), and (**D**) Stability of phage LPST94 in four food samples (milk, apple juice, chicken breast, and lettuce) at 25 °C. Values represent mean with standard deviation of three determinations of each point.

The characteristics of phage LPST94 are shown in Figure 3. According to the one-step growth curve, the latent period was approximately 10 min and the average burst size was 145 ± 10 PFU/cell (Figure 3A). This phage is very stable with a pH range from 4 to 12, however, phage titers were reduced when the pH was in the higher (pH > 12) or lower (pH < 4) ranges (Figure 3B). The phage had a high degree of thermal tolerance, with stability as high as 60 °C but reduced at 70 °C and 80 °C (Figure 3C). By incubating phage within food matrices including milk, chicken breast, and lettuce the phage showed either no difference or minimal titer reduction within the experimental timescale of 48 h. However, the phage titer fell by approximately 2.5 log PFU/mL after 48 h incubation in apple juice (Figure 3D). Lysogenic induction assay results showed that LPST94 phage-resistant *Salmonella* were unable to lysogens (Figure S1). In summary, phage LPST94 remained stable under all test conditions described above and may be a promising candidate to control *Salmonella* in foods.

### 3.5. Application of Phage LPST94 in Controlling Food-Borne S. Typhimurium and S. Enteritidis

*Salmonella* attributed outbreaks are often associated with animal products including poultry, raw meat, dairy production, but also include products such as salad dressing and fruit juice [51]. In this study, the efficiency of phage LPST94 was assayed in different food matrices against *S.* Typhimurium and *S.* Enteritidis.

#### 3.5.1. Milk

**Figure 4:**
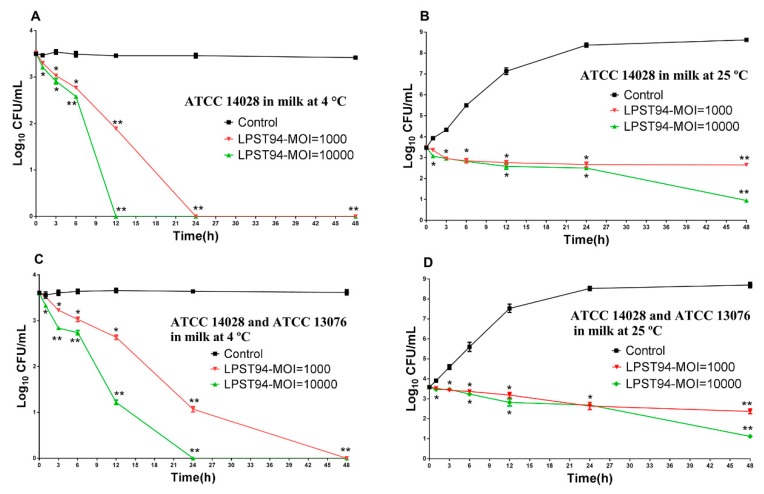
Effectiveness of phage LPST94 in reducing the *S.* Typhimurium ATCC 14028 and *S.* Enteritidis ATCC 13076 in milk. (**A**) Effect of phage LPST94 on growth of *S.* Typhimurium ATCC 14,028 in milk at 4 °C, (**B**) Effect of phage LPST94 on growth of *S.* Typhimurium ATCC 14028 in milk at 25 °C, (**C**) Effect of phage LPST94 on growth of *Salmonella* mixture (*S.* Typhimurium ATCC 14028 and *S.* Enteritidis ATCC 13076) in milk at 4 °C, and (**D**) Effect of phage LPST94 on growth of *Salmonella* mixture (*S.* Typhimurium ATCC 14028 and *S.* Enteritidis ATCC 13076) in milk at 25 °C. Values represent mean with standard deviation of three determinations. ** Significance of *p* < 0.01; * Significance of *p* < 0.05.

Milk was inoculated with *S.* Typhimurium (ATCC 14028) alone or a mixture of *Salmonella* (*S.* Typhimurium ATCC 14028 and *S.* Enteritidis ATCC 13076) to a final viable count of 3.5 log_10_ CFU/mL. After incubation with phage LPST94, the viable count of the *S.* Typhimurium in milk was reduced below the detection limit (<1 CFU/100 μL) after 12 h and 24 h at 4 °C using an MOI of 10,000 and 1000, respectively (Figure 4A). At 25 °C after 48 h incubation, the viable count of *Salmonella* decreased from 3.5 log_10_ CFU/mL down to 2.56 and 0.84 log_10_ CFU/mL with phage added to an MOI of 10,000 and 1000, respectively (Figure 4B). Similar results were observed when using phage LPST94 to infect the mixture of *Salmonella* (*S.* Typhimurium ATCC 14028 and *S.* Enteritidis ATCC 13076). There was almost complete elimination of viable bacteria in milk after 24 h and 48 h at 4 °C with an MOI of 10,000 and 1000 (Figure 4C). Biocontrol by LPST94 resulted in decreasing viable *Salmonella* counts of at least 2.48 log_10_ CFU/mL and 1.22 log_10_ CFU/mL with an MOI of 10,000 and 1000 at 25 °C, respectively (Figure 4D).

#### 3.5.2. Apple Juice

**Figure 5:**
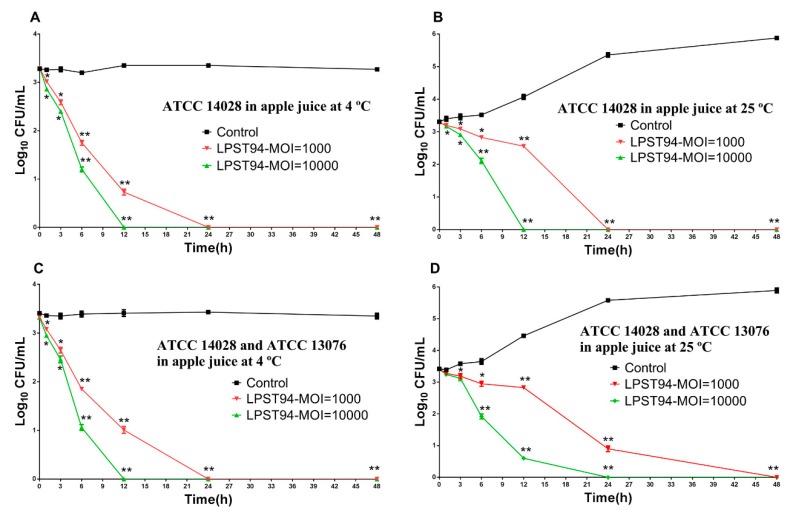
Effectiveness of phage LPST94 in reducing the *S.* Typhimurium ATCC 14028 and *S.* Enteritidis ATCC 13076 in apple juice. (**A**) Effect of phage LPST94 on growth of *S.* Typhimurium ATCC 14028 in apple juice at 4 °C, (**B**) Effect of phage LPST94 on growth of *S.* Typhimurium ATCC 14028 in apple juice at 25 °C, (**C**) Effect of phage LPST94 on growth of *Salmonella* mixture (*S.* Typhimurium ATCC 14028 and *S.* Enteritidis ATCC 13076) in apple juice at 4 °C, and (**D**) Effect of phage LPST94 on growth of *Salmonella* mixture (*S.* Typhimurium ATCC 14028 and *S.* Enteritidis ATCC 13076) in apple juice at 25 °C. Values represent mean with standard deviation of three determinations. ** Significance of *p* < 0.01; * Significance of *p* < 0.05.

Similar to the milk model, apple juice was artificially inoculated with *S.* Typhimurium (ATCC 14028) alone or as a mixture of *Salmonella* (*S.* Typhimurium ATCC 14028 and *S.* Enteritidis ATCC 13076) to 3 log_10_ CFU/mL. Relative to the non-treated controls, the viable *Salmonella* counts (*S.* Typhimurium) were reduced by using LPST94 at an MOI of 10,000 and 1000. When incubated at 4 °C, the addition of LPST94 at an MOI of 10,000 and 1000, resulted in the *Salmonella* viable counts falling below the detection threshold after 12 h and 24 h, respectively (Figure 5A). Similar results were also observed when samples were incubated at 25 °C, where the *Salmonella* counts were eliminated after 12 h and 24 h upon application of phage at an MOI of 10,000 and 1000, respectively (Figure 5B). Figure 5C shows that when phage LPST94 was applied at 4 °C against a mixture of *S.* Typhimurium ATCC14028 and ATCC13076 with an MOI of 10,000 and 1000, viable count of the bacteria was significantly reduced to undetectable levels after 12 h and 24 h respectively, which was consistent with the single *Salmonella* strain model. However, when LPST94 was applied to the *Salmonella* host mixture at 25 °C, the phage anti-bacterial efficiency was comparatively impaired, since the time to reach undectable levels were increased to 24 h and 48 husing the similar MOIs of 10,000 and 1000 (Figure 5D).

#### 3.5.3. Chicken Breast

**Figure 6:**
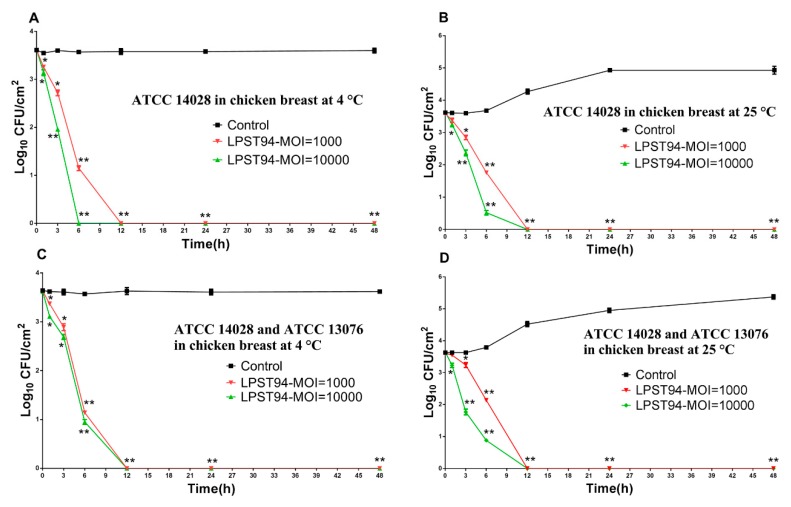
Effectiveness of phage LPST94 in reducing the *S.* Typhimurium ATCC 14028 and *S.* Enteritidis ATCC 13076 in chicken breast. (**A**) Effect of phage LPST94 on growth of *S.* Typhimurium ATCC 14028 on chicken breast at 4 °C, (**B**) Effect of phage LPST94 on growth of *S.* Typhimurium ATCC 14028 on chicken breast at 25 °C, (**C**) Effect of phage LPST94 on growth of *Salmonella* mixture (*S.* Typhimurium ATCC 14028 and *S.* Enteritidis ATCC 13076) on chicken breast at 4 °C, and (**D**) Effect of phage LPST94 on growth of *Salmonella* mixture (*S.* Typhimurium ATCC 14028 and *S.* Enteritidis ATCC 13076) on chicken breast at 25 °C. Values represent mean with standard deviation of three determinations. ** Significance of *p* < 0.01; * Significance of *p* < 0.05.

Phage LPST94 conferred an appreciable reduction of viable *Salmonella* counts on chicken breasts at both 4 °C and 25 °C with MOIs of 10,000 and 1000 (Figure 6). When administered at an MOI of 10,000, phage LPST94 reduced viable *Salmonella* below the level of detection at 4 °C after 6 h (Figure 6A) and at 25 °C after 12 h of incubation (Figure 6B). In the case of an MOI of 1000 inoculation, LPST94 eliminated surviving *Salmonella* below detectable limits after 12 h of incubation at both at 4 °C (Figure 6A) and 25 °C (Figure 6B). The mixture of *Salmonella* was also eliminated with MOIs of 10,000 and 1000 at both 4 °C (Figure 6C) and 25 °C (Figure 6D), although, the incubation period for this complete reduction was 12 h for each.

#### 3.5.4. Lettuce

**Figure 7:**
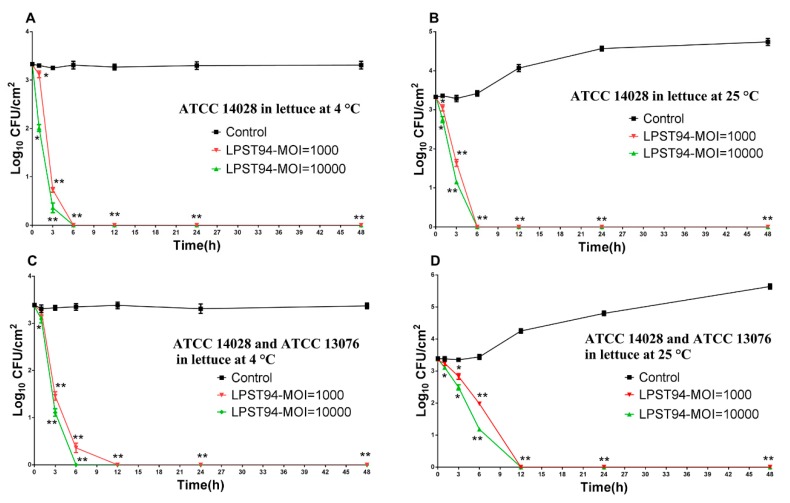
Effectiveness of phage LPST94 in reducing the *S.* Typhimurium ATCC 14028 and *S.* Enteritidis ATCC 13076 in lettuce. (**A**) Effect of phage LPST94 on growth of *S.* Typhimurium ATCC 14028 on lettuce at 4 °C, (**B**) Effect of phage LPST94 on growth of *S.* Typhimurium ATCC 14028 on lettuce at 25 °C, (**C**) Effect of phage LPST94 on growth of *Salmonella* mixture (*S.* Typhimurium ATCC 14028 and *S.* Enteritidis ATCC 13076) on lettuce at 4 °C, and (**D**) Effect of phage LPST94 on growth of *Salmonella* mixture (*S.* Typhimurium ATCC 14028 and *S.* Enteritidis ATCC 13076) on lettuce at 25 °C. Values represent mean with standard deviation of three determinations. ** Significance of *p* < 0.01; * Significance of *p* < 0.05.

The ability of the phage to reduce the artificial *Salmonella* contamination of lettuce was also demonstrated, with reductions below the level of detection for *Salmonella* at 4 °C and 25 °C after 6 h using an MOI of 10,000 and 1000, respectively (Figure 7A,B). The effect of phage against a mixture of *Salmonella* on lettuce was comparable with no viable *Salmonella* observed at 4 °C after 6 h and 12 h using an MOI of 10,000 and 1000, respectively (Figure 7C). Likewise, there was a complete reduction of the mixture of *Salmonella* at 25 °C after 12 h (Figure 7D).

## 4. Discussion

In this study, 40 putative different phages were isolated from the environmentally sourced water samples and screened to identify a novel broadly acting phage against *Salmonella* species [52,53]. Based on lytic spectrum analysis, phage LPST94 showed a wide lytic range and related lytic activity against all 11 *Salmonella* serovars tested including *S.* Typhimurium, *S.* Enteritidis, *S.* Pullorum, *S.* Dublin, *S.* Anatum, *S.* Arizonae, *S.* Javiana, *S.* Kentucky, *S.* Newport, *S.* Paratyphi B, and *S.* Choleraesuls. Interestingly, phage LPST94 could also lyse multi-drug-resistant *Salmonella.* Clear plaques were observed for all 18 multi-drug-resistant *Salmonella* strains tested after phage LPST94 infection.

Phage LPST94 had high lytic activity against all the *Salmonella* in vitro and could inhibit the growth of 4 *Salmonella* (*S.* Typhimurium UK-1, *S.* Typhimurium ATCC 14028, *S.* Enteritidis ATCC 13076 and *S.* Enteritidis SGSC4901) for up to 11 h at MOIs of 0.1, 1, 10, and 100. In comparison, the phage FGCSSa1 was reported to inhibit growth of their host (*S.* Typhimurium PT160) for up to 2 h at MOIs of 0.3 and 2.5 [52].

Transmission electron microscopy and genome analysis confirmed that phage LPST94 belonged to the *Ackermannviridae* family. Phage morphology with long tails were considered as professionally lytic phage types [54], which can be used in phage-based antibacterial strategies [55]. Phage LPST94 was robust, exhibiting stability over the pH range 4 to 12 and able to maintain lysis activity at 60 °C for at least 60 min. The pH and thermal stability of phage LPST94 indicate that it may be applied for biocontrol over a variety of conditions employed in food processing. This study showed that phage remained stable over 48 h in milk, chicken breast, and lettuce but 2.5 log_10_ PFU/mL losses were observed in apple juice potentially due to presence of weak acid. However, small losses in phage titers in diverse food samples (Chinese cabbage, chicken breast, mixed seafood, and chocolate milk) have been observed by other researches [28,47]. These results suggest that phage LPST94 has clear potential to be applied on raw and processed food samples.

We further determined the efficiency of this phage to reduce the counts of *Salmonella* within foods. In experiments with milk, phage LPST94 reduced viable *Salmonella* counts 3 log_10_ at 4 °C and 2.56 log_10_ CFU/mL at 25 °C after 48 h incubation, which was of greater efficiency when compared with phage PA 13076 that was reported to reduce *S.* Enteritidis ATCC13076 by 1 log_10_ CFU/mL at 4 °C and 25 °C after 5 h incubation in milk [28]. Within apple juice, phage LPST94 reduced *Salmonella* by 3 log_10_ at 4 °C after 12 h incubation using an MOI of 10,000, similar to that reported for phage P22 [56]. In chicken breast and lettuce, *Salmonella* counts dropped below detectable levels (<1 CFU/100 μL) at 4 °C and 25 °C from 6 h to 12 h of incubation. This may be compared with a 3 log_10_ reduction in the *Salmonella* viable count reported after phage treatment for Chinese cabbage [28], 1.7 log_10_ reduction for lettuce [26], 1.37 log_10_ for mustard and 0.55 log_10_ reduction for broccoli [57].

Genomic sequence analysis of phage LPST94 revealed similarity to phage PhiSH19 but with novel features that confer prospective functions for infection and proliferation. Phage LPST94 genome contained genes for structure and replication/recombination/repair, nucleotide metabolism, transcription, translation and additional functions for phage. These results indicated that this phage could replicate efficiently but was unable to form a lysogen in *Salmonella* [58,59,60]. In addition, a feature of phage LPST94 was that it encoded several different tail spike proteins which are hypothesized as an indicator of a broad lytic range [61]. The tail spikes primary recognize, attach, and cleave the LPS O-antigen of *S.* Typhimurium [62]. Moreover, the phage genome did not encode any virulence genes, toxins, drug-resistant markers or integration genes, indicating that this phage has the potential for being developed as an alternative antimicrobial agent without any harmful effects on humans and animals.

## 5. Conclusions

We have isolated a novel broad host lytic phage LPST94 against diverse *Salmonella* serovars. Phage LPST94 exhibited tolerance against a range pH, thermal conditions, and stability for 48 h upon incubation with different food samples, properties that are a prerequisite for a biocontrol agent that can be directly applied in food samples. Further study showed this phage can effectively control *Salmonella* strains in a variety of food samples (milk, apple juice, chicken breast, and lettuce), suggesting phage LPST94 is a prime candidate for the biological control of *Salmonella*.

## Figures and Tables

**Table 1:** Sensitivity of different *Salmonella* serovars and other bacterial strains against selected phages determined by spot testing.

Bacterial Strains		% of Positive Spot Test Against *Salmonella* Serovars and Other Bacterial Strains							
	LPSTSA	LPSTSD	LPSTSF	LPST94	LPSTSH	LPSTSK	LPSTSN	LPSTSP	LPSTSV
*Salmonella* serovars									
Typhimurium (*N* = 7)	85.7	100	100	100	85.7	71.4	71.4	85.7	85.7
Enteritidis (*N* = 5)	80	60	40	100	80	60	80	60	80
Dublin (*N* = 2)	50	100	100	100	50	100	50	100	50
Choleraesuls (*N* = 1)	0	100	0	100	100	0	0	100	100
Newport (*N* = 1)	100	0	100	100	0	0	100	0	0
Paratyphi B (*N* = 1)	100	100	0	100	100	100	0	100	100
Anatum (*N* = 1)	0	0	100	100	0	0	0	100	0
Pullorum (*N* = 1)	0	0	0	100	0	100	100	0	100
Javiana (*N* = 1)	0	100	100	100	0	0	100	0	100
Kentucky (*N* = 1)	100	0	100	100	100	0	100	100	0
*S. arizonae* (*N* = 1)	0	100	0	100	0	100	0	100	100
Drug-resistant *Salmonella* serovars									
Typhimurium (*N* = 10)	60	80	90	100	80	70	90	80	90
Enteritidis (*N* = 8)	37.5	62.5	50.0	100	62.5	50.0	62.5	87.5	37.5
Other bacterial strains									
*E. coli* (*N* = 6)	0	0	0	0	0	0	0	0	0
*A. hydrophila* (*N* = 4)	0	0	0	0	0	0	0	0	0
*C. sakazakii* (*N* = 3)	0	0	0	0	0	0	0	0	0
*S. flexneri* (*N* = 1)	0	0	0	0	0	0	0	0	0
*V. parahaemolyticus* (*N* = 2)	0	0	0	0	0	0	0	0	0
*P. aeruginosa* (*N* = 1)	0	0	0	0	0	0	0	0	0
*S. aureus* (*N* = 3)	0	0	0	0	0	0	0	0	0
*Listeria* (*N* = 2)	0	0	0	0	0	0	0	0	0
*S. Suis* (*N* = 2)	0	0	0	0	0	0	0	0	0
*L. acidophilus* (*N* = 1)	0	0	0	0	0	0	0	0	0

**Table 2:** Efficiency of plating (EOP) by phage LPST94 against different *Salmonella* serovars.

Bacterial Strains	EOP of LPST94	Drug-Resistance *Salmonella*	EOP of LPST94
*S.Enterica* serovar Typhimurium		*S.Enterica* serovar Typhimurium	
ATCC 14028	+++	LST10	+
ATCC 13311	+++	LST11	++
UK-1	+++	LST12	++
ST8	+++	LST13	++
SGSC 4903	+++	LST14	+
SL 1344	+++	LST15	+
LT2	+++	LST16	+
*S.enterica* serovar Enteritidis		LST17	++
ATCC 13076	++	LST18	+
SJTUF 10978	+	LST19	+
SJTUF 10984	+	*S.enterica* serovar Enteritidis	
LK5-3820	++	LSE6	+
SGSC 4901	++	LSE7	+
*S.enterica* serovar Dublin		LSE8	++
3710	+	LSE9	++
3723	+	LSE10	+
*S. enterica* serovar Choleraesuis		LSE11	+
ATCC 10708	+	LSE12	+
*S. enterica* serovar Newport		LSE15	+
E20002725	+		
*S. enterica* serovar Paratyphi B			
CMCC 50094	++		
*S. enterica* Serovar Pullorum			
CVCC 519	+		
*S. enterica* serovar Javiana			
CVM 35943	+		
*S. enterica* serovar Anatum			
ATCC 9270	+		
*S. enterica* serovar Kentucky			
CVM 29188	+		
*S. enterica* Arizonae			
CDC 346-86	+		

+++, EOP 0.5 to 1.0; ++, EOP 0.2 to <0.5; +, 0.001 to <0.2.

## Supplementary Materials

The following are available online at https://www.mdpi.com/2076-2607/8/2/247/s1. Figure S1. Confirmation of LPST94 was unable to form lysogen. Induced by mitomycin C, found to contain lysogenic phage. Table S1. List of bacterial strains used in this study. Table S2. Functional annotation of LPST94 CDSs using BLASTP and conserved domains. Table S3. Comparison of phages against the LPST94 genome. Table S4. Antibiotics resistance profiles of the clinical isolates of Salmonella.
