# Supplementary material for: Application of a Broad Range Lytic Phage LPST94 for Biological Control of Salmonella in Foods
Source: Microorganisms. 2020 Feb 13;8(2):247. doi: 10.3390/microorganisms8020247 (PMC7074677; doi:10.3390/microorganisms8020247)
Supplement: Supplementary file 1 [file microorganisms-08-00247-s001.pdf]

microorganisms

Supplementary Materials

Application of a broad range lytic phage LPST94 for biological control of *Salmonella* in foods

Md. Sharifull Islam[1,2,3], Yang Zhou[3,4], Lu Liang [5], Ishatur Nime[1], Ting Yan[1], Stephan P. Willias[6], Md. Zakaria Mia[7], Weicheng Bei[2,3], Ian F. Connerton[5], Vincent A. Fischetti[8], Jinquan Li[1,3,8]*

[1] Key Laboratory of Environment Correlative Dietology, College of Food Science and Technology, Huazhong Agricultural University, Wuhan, Hubei, People's Republic of China; smbgb101287@yahoo.com (M.S.I); smbgb101287@gmail.com (I.N); yantingau@163.com (T.Y.); lijinquan2007@gmail.com (J. Li)

[2] College of Veterinary Medicine, Huazhong Agricultural University, Wuhan 430070, PR China; beiwe@mail.hzau.edu.cn (W.B)

[3] State Key Laboratory of Agricultural Microbiology, Huazhong Agricultural University, Wuhan, Hubei, People's Republic of China;

[4] College of Fisheries, Huazhong Agricultural University, Wuhan, Hubei, People's Republic of China; zhouyang@ mail.hzau.edu.cn (Y.Z)

[5] Division of Food Sciences, University of Nottingham, Sutton Bonington Campus, 11 Loughborough, Leicestershire, United Kingdom; sbzll3@nottingham.ac.uk (L.L); scziac@exmail.nottingham.ac.uk (I.F.C)

[6] Department of Infectious Diseases and Immunology, University of Florida, Gainesville, Florida, United States; swillias@ehs.ufl.edu (S.P.W)

[7] Department of Microbiology, Jagannath University, Dhaka, Bangladesh; mmzakaria@yahoo.com (M.Z.M)

[8] Laboratory of Bacterial Pathogenesis and Immunology, The Rockefeller University, New York, New York, USA
lijinquan2007@gmail.com (J. Li); vaf@mail.rockefeller.edu (V.A.F)

* Correspondence: lijinquan2007@gmail.com; lijinquan@mail.hzau.edu.cn (J. Li)

1. Ability of Lysogen Formation of the Phage

As shown in Supplementary Fig. 1, *Salmonella enterica* serovar Enteritidis ATCC 13076 and SGSC 4901 had prophage, while *Salmonella enterica* serovar Typhimurium ATCC 13311 had no prophage. Thus, we selected phage-resistant single colony of ATCC13311 to determine the lysogenicity of LPST94. The result showed that no phage was detected in the supernatant of phage-resistant *Salmonella enterica* serovar Typhimurium ATCC 13311 after treatment with mitomycin C (1 μg/mL), this results proved that LPST94 was unable to form lysogen.

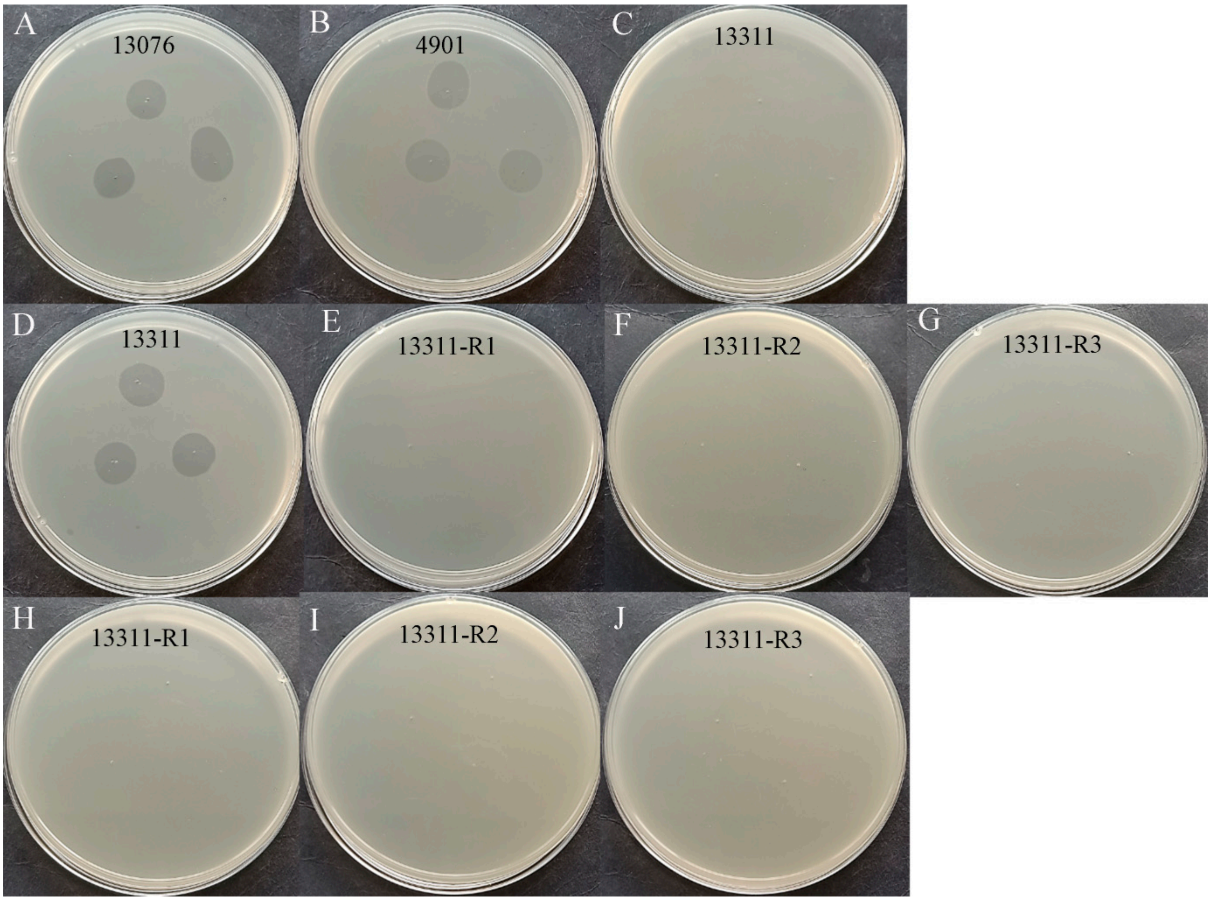

Figure S1. Confirmation of LPST94 was unable to form lysogen. Induced by mitomycin C, found to contain lysogenic phage (A) *Salmonella enterica* serovar Enteritidis ATCC 13076, (B) *Salmonella enterica* serovar Enteritidis SGSC 4901, while not found to contained lysogenic phage (C) *Salmonella enterica* serovar Typhimurium ATCC 13311. (D) Spot test results confirm that LPST94 could lyse *Salmonella enterica* serovar Typhimurium ATCC 13311 and could be used for generation of phage-resistant host strain and lysogenic phage induction experiment. Three phage-resistant ATCC 13311 strains, (E) 13311-R1, (F) 13311-R2, and (G) 13311-R3, were confirmed to be phage LPST94 resistant by spot test. Three phage-resistant strains were induced with mitomycin C respectively, and no phages were detected by spot test (H) 13311-R1, (I) 13311-R2, and (J) 13311-R3.

Table S1. List of bacterial strains used in this study.

Bacterial Strains	Strain ID number	Numbers of strains	Source of strains
S. enterica serovar Typhimurium	ATCC 14028, ATCC 13311	2	ATCC
	SGSC 4903	1	SGSC
	LST2 (ST8), LST4 (UK-1), LST6 (LT2), LST8 (SL1344)	4	LS
S. enterica serovar Enteritidis	ATCC 13076	1	ATCC
	SJTUF 10978, SJTUF 10984	2	SJTU
	SGSC 4901	1	SGSC
	LSE4 (LK5-3820)	1	LS
S. enterica serovar Pullorum	LSP1 (CVCC 519)	1	LS
S. enterica serovar Dublin	LSD1 (3710), LSD2 (3723)	2	LS
S. enterica serovar Anatum	ATCC 9270	1	ATCC
S. enterica Arizonae	CDC 346-86	1	CDC
S. enterica serovar Javiana	LSX23 (CVM 35943)	1	LS
S. enterica serovar Kentucky	LSX24 (CVM 29188)	1	LS
S. enterica serovar Newport	E20002725	1	CDC
S. enterica serovar Paratyphi B	CMCC 50094	1	CMCC
S. enterica serovar Choleraesuls	ATCC 10708	1	ATCC
Drug resistant *S. enterica* serovar Typhimurium	LST10, LST11, LST12, LST13, LST14, LST15, LST16, LST17, LST18, LST19	10	LS
Drug resistant *S. enterica* serovar Enteritidis	LSE6, LSE7, LSE8, LSE9, LSE10, LSE11, LSE12, LSE15	8	LS
E. coli	BL21, DH5α	2	TB
	ATCC 933	1	ATCC
	LEC1 (F18AC), LEC2 (C83715), LEC3 (T10)	3	LS
A. hydrophila	ZYAH72, ZYAH75, J1, ZYAH91 (D4)	4	LS
C. sakazakii	ATCC 12868, ATCC 29004, ATCC 29544	3	ATCC
S. flexneri	CMCC 51572	1	CMCC
V. parahaemolyticus	ATCC 17802, ATCC 33846	2	ATCC
P. aeruginosa	ATCC 7853	1	ATCC
S. aureus	ATCC 6538, ATCC 8095, ATCC 29213	3	ATCC
Listeria	ATCC 19114, ATCC 19115	2	ATCC
Streptococcus suis	LSM122 (P1/7), LSM123 (SC19)	2	LS
L. acidophilus	ATCC SD5221	1	ATCC

Abbreviation: ATCC, American Type Culture Collection; SGSC, *Salmonella* Genetic Stock Center; LS, Lab Stock; SJTU, Shanghai Jiao Tong University; CDC, Centers for Disease Control and Prevention; TB, TransGen Biotech; CMCC, National Center for Medical Culture Collection.

Table S2. Functional annotation of LPST94 CDSs using BLASTP and conserved domains

ORF	Gene Positions	Functions	Name	Accession
**1**	1746-2054	hypothetical protein [Salmonella phage vB_SalM_SJ2]	PHA02092 super family	cl10367
**2**	2051-2380	DUF4326 domain-containing protein [Alicyclobacillus sendaiensis]	DUF4326	pfam14216
**3**	2361-2645	hypothetical protein [Escherichia phage PhaxI]		
**4**	2742-3359	RegB endoribonuclease [Salmonella phage Marshall]		
**5**	3359-3997	hypothetical protein [Escherichia phage ECML-4]		
**6**	4102-4377	hypothetical protein DET7_72 [Salmonella phage Det7]		
**7**	4443-6902	hypothetical protein [Salmonella phage SP1]		
**8**	6954-7523	hypothetical protein [Salmonella phage vB_SalM_SJ2]		
**9**	7583-7936	hypothetical protein [Salmonella phage vB_SalM_SJ3]	rI.-1 super family	cl14362
**10**	8000-8617	hypothetical protein DET7_76 [Salmonella phage Det7]		
**11**	8617-9681	DNA primase [Salmonella phage vB_SalM_SJ3]	61 super family	cl26791
**12**	9678-9887	hypothetical protein [Salmonella phage vB_SalM_SJ3]		
**13**	10087-10575	hypothetical protein [Escherichia phage PhaxI]		
**14**	10626-10814	hypothetical protein [Salmonella phage vB_SalM_SJ2]		
**15**	10884-11678	endolysin [Salmonella phage Mutine]	DUF3380	pfam11860
**16**	11786-12625	PhoH-like phosphate starvation-inducible protein [Salmonella virus ViI]	P-loop_NTPase super family	cl21455
**17**	12710-14986	ribonucleotide reductase of class Ia, alpha subunit [Salmonella phage SP1]	Ribonuc_red_lgC super family	cl27341
**18**	15057-16160	ribonucleotide-diphosphate reductase beta subunit [Salmonella phage Mutine]	Ferritin_like super family	cl00264
**19**	16170-16394	Glutaredoxin [Salmonella virus ViI]	Thioredoxin_like super family	cl00388
**20**	16428-16733	hypothetical protein [Salmonella phage vB_SalM_SJ2]		
**21**	16745-17196	hypothetical protein [Escherichia phage ECML-4]		
**22**	17198-17536	hypothetical protein [Salmonella phage vB_SalM_PM10]		
**23**	17737-17917	baseplate wedge protein [Salmonella phage Marshall]	GPW_gp25 super family	cl01403

**24**	17982-19607	baseplate hub subunit with lysozyme motif [Salmonella phage Det7]	Gp5_OB super family	cl28617
**25**	20114-20920	baseplate hub subunit [Salmonella phage S8]	T4_baseplate super family	cl27821
**26**	20970-21494	hypothetical protein [Salmonella phage SP1]		
**27**	21472-21957	hypothetical protein S8_046 [Salmonella phage S8]		
**28**	21995-22606	RuvC-like endodeoxyribonuclease [Klebsiella virus vB_KpnM_KpS110]		
**29**	22599-22895	putative membrane protein [Salmonella phage vB_SalM_PM10]		
**30**	22882-23127	regulatory protein [Salmonella phage Sh19]	CxxC_CXXC_SSSS	smart00834
**31**	23120-23362	Gp33 late promoter transcription factor [Salmonella virus ViI]	Trans_coact super family	cl25123
**32**	23372-23611	hypothetical protein SP38_192 [Salmonella phage 38]		
**33**	23708-24739	single stranded DNA-binding protein [Salmonella phage GG32]	gp32 super family	cl17537
**34**	24767-25711	baseplate tail tube [Salmonella phage Sh19]		
**35**	25766-26470	Gp2 DNA end protector protein [Salmonella virus ViI]	2	PHA02577
**36**	26532-27278	hypothetical protein [Salmonella phage vB_SalM_PM10]		
**37**	27296-27583	hypothetical protein [Salmonella phage vB_SalM_PM10]		
**38**	27757-28716	hypothetical protein DET7_106 [Salmonella phage Det7]	Neuromodulin_N super family	cl26511
**39**	28789-29451	baseplate hub [Salmonella phage Det7]	Thymidylate_kin super family	cl27056
**40**	29451-30491	thymidylate synthase [Salmonella phage Sh19]	TS_Pyrimidine_HMase super family	cl19097
**41**	30488-31057	putative deoxynucleotide monophosphate kinase [Salmonella phage GG32]		
**42**	31054-31605	putative dUTP diphosphatase [Salmonella phage S8]	NTP-PPase super family	cl16941
**43**	31605-32135	hypothetical protein DET7_111 [Salmonella phage Det7]		
**44**	32120-33205	RecA-like recombination protein [Salmonella phage FSL SP-063]	RecA	COG0468
**45**	33183-33512	hypothetical protein [Salmonella phage vB_SalM_SJ2]		
**46**	33519-34943	DNA primase-helicase subunit [Salmonella phage vB_SalM_SJ3]	DnaB_C super family	cl27163
**47**	35007-35339	putative GTPase-activator protein [Serratia phage phiMAM1]		
**48**	35352-35654	GTPase-activator protein [Serratia phage 2050H1]		

**49**	35772-36965	hypothetical protein [Salmonella phage SP1]		
**50**	35962-37078	hypothetical protein Marshall_158 [Salmonella phage Marshall]		
**51**	37078-37287	DNA ligase [Serratia phage phiMAM1]		
**52**	37289-37627	hypothetical protein SP063_00395 [Salmonella phage FSL SP-063]		
**53**	37689-39113	DNA ligase [Salmonella phage S8]	CDC9 super family	cl25417
**54**	39191-39454	hypothetical protein [Salmonella phage GG32]		
**55**	39598-40260	Gp59 Loader of T4-like helicase [Salmonella virus ViI]	T4_Gp59_N super family	cl27869
**56**	40261-42201	putative tape measure protein [Salmonella phage Mutine]		
**57**	42212-43600	putative baseplate hub subunit gp27 [Enterobacter phage phiEM4]		
**58**	43587-44153	baseplate wedge subunit [Salmonella phage Sh19]	Phage_gp53 super family	cl26475
**59**	44167-45135	Gp48 T4-like baseplate tail tube cap [Salmonella virus ViI]		
**60**	45189-45809	head completion protein [Salmonella phage 38]	Tn7_Tnp_TnsA_N super family	cl21695
**61**	46020-46427	pleckstrin homology (PH containing protein [Salmonella phage Marshall]	bPH_2	pfam03703
**62**	46429-46947	dCMP deaminase [Salmonella phage Sh19]	cytidine_deaminase-like super	cl00269
**63**	46940-47347	MULTISPECIES: aldehyde dehydrogenase [Streptomyces]		
**64**	47344-47685	hypothetical protein BSP101_0010 [Salmonella phage BSP101]		
**65**	47749-48099	hypothetical protein [Salmonella phage vB_SalM_SJ3]		
**66**	48096-48428	hypothetical protein BSP101_0012 [Salmonella phage BSP101]		
**67**	48425-48985	putative serine/threonine protein phosphatase [Salmonella phage S8]	MPP_superfamily super family	cl13995
**68**	48985-49326	hypothetical protein [Salmonella phage vB_SalM_SJ3]		
**69**	49329-49991	putative alpha hydrolase [Erwinia phage phiEa2809]	AANH_like super family	cl00292
**70**	50089-50379	hypothetical protein vB_SenM-2_136 [Salmonella phage vB_SenM-2]		
**71**	50457-51089	exonuclease [Salmonella phage vB_SenM-2]	DnaQ_like_exo super family	cl10012
**72**	51082-51426	hypothetical protein CPT_Mutine_203 [phage Mutine]		
**73**	51423-52028	Tk.4 protein [Shigella phage Ag3]	Macro super family	cl00019

**74**	52025-52261	hypothetical protein [Salmonella phage vB_SalM_PM10]		
**75**	52258-52482	hypothetical protein SP063_00260 [Salmonella phage FSL SP-063]		
**76**	52532-52945	tRNA processing enzyme [Klebsiella virus vB_KpnM_KpS110]	nitrilase super family	cl11424
**77**	52949-53260	anaerobic dehydrogenase [Acinetobacter proteolyticus]		
**78**	53262-53540	putative membrane protein [Salmonella phage vB_SalM_SJ3]		
**79**	53583-54914	DNA topoisomerase II [Salmonella phage Det7]	TOP4c super family	cl27672
**80**	54916-56817	DNA topoisomerase II [Salmonella phage Sh19]	TOP2c super family	cl25574
**81**	56810-57640	putative HNH homing endonuclease domain protein [Dickeya phage phiD3]	HNHc	cd00085
**82**	57637-58221	viral tegument-like protein [Salmonella phage vB_SalM_SJ2]		
**83**	58218-58703	hypothetical protein DET7_154 [Salmonella phage Det7]		
**84**	58744-58935	hypothetical protein DET7_155 [Salmonella phage Det7]		
**85**	58983-59486	putative histone-like protein [Shigella phage Ag3]		
**86**	59575-59796	TonB-linked outer membrane protein [Chryseobacterium sp. Leaf201]		
**87**	59800-60609	tail fiber protein [Salmonella phage vB_SalM_SJ2]	ChW super family	cl02763
**88**	60588-60980	hypothetical protein vB_SenM-2_153 [Salmonella phage vB_SenM-2]	PHA02335 super family	cl10423
**89**	61013-61426	hypothetical protein [Salmonella phage vB_SalM_SJ3]		
**90**	61401-61700	hypothetical protein Maynard_199 [Salmonella phage Maynard]		
**91**	61754-63337	rIIB [Salmonella phage Maynard]	HTH super family	cl21459
**92**	63370-66111	RIIA [Salmonella phage Sh19]		
**93**	66219-66572	hypothetical protein [Salmonella phage vB_SalM_SJ3]		
**94**	66742-66948	hypothetical protein DET7_165 [Salmonella phage Det7]		
**95**	66941-67390	hypothetical protein [Salmonella phage vB_SalM_PM10]		
**96**	67387-67758	cytochrome c maturation protein CcmE [Ensifer adhaerens]	PRK06847 super family	cl27550
**97**	67820-68242	hypothetical protein Marshall_6 [Salmonella phage Marshall]		
**98**	68247-68816	major capsid protein [Pectobacterium phage DU_PP_V]	HAD_like super family	cl21460

**99**	68832-69941	putative membrane protein [Pectobacterium phage CBB]	TerC super family	cl10468
**100**	69938-70324	hypothetical protein BSP101_0047 [Salmonella phage BSP101]		
**101**	70321-70533	hypothetical protein [Dickeya virus Limestone]		
**102**	70545-70736	hypothetical protein CPT_Merlin119 [Citrobacter phage Merlin]		
**103**	70818-70985	hypothetical membrane protein [Shigella phage Ag3]		
**104**	71022-72257	hypothetical protein S8_126 [Salmonella phage S8]		
**105**	22254-72439	hypothetical protein [Salmonella phage SKML-39]		
**106**	72420-72806	hypothetical protein [Salmonella phage SP1]		
**107**	72808-73011	hypothetical protein DET7_178 [Salmonella phage Det7]		
**108**	73014-73919	hypothetical protein [Salmonella phage SP1]		
**109**	73919-74224	putative thioredoxin [Dickeya phage JA15]		
**110**	74235-75017	deoxyribonucleotidase [Salmonella phage Marshall]	HAD_like super family	cl21460
**111**	75010-75372	putative lipoprotein [Klebsiella phage May]		
**112**	75433-77058	DNA polymerase [Salmonella phage vB_SalM_SJ3]	POLBc super family	cl10023
**113**	77172-78050	putative intron I-LimII [Dickeya virus Limestone]		
**114**	78778-70611	DNA polymerase [Salmonella phage SKML-39]	POLBc super family	cl10023
**115**	79673-80263	hypothetical protein STP07_019 [Salmonella phage STP07]		
**116**	80614-81171	hypothetical protein SP38_32 [Salmonella phage 38]		
**117**	81220-81762	hypothetical protein DET7_187 [Salmonella phage Det7]		
**118**	81757-82133	hypothetical protein STP07_014 [Salmonella phage STP07]		
**119**	82159-82617	hypothetical protein [Salmonella phage vB_SalM_SJ3]		
**120**	82653-83102	hypothetical protein [Salmonella phage vB_SalM_SJ3]	DUF3990 super family	cl16135
**121**	83142-84335	pyridoxal-phosphate dependent enzyme [Salmonella phage Maynard]	PALP super family	cl25429
**122**	84405-85106	hypothetical protein S8_144 [Salmonella phage S8]	ETC_C1_NDUFA4 super family	cl04760
**123**	85178-85693	tail needle and knob protein [Pectobacterium phage PP99]	hage_tail_NK super family	cl24937

**124**	85704-85940	hypothetical protein [Salmonella phage vB_SalM_PM10]		
**125**	89735-90304	hypothetical protein [Salmonella phage vB_SalM_PM10]		
**126**	90668-92449	baseplate wedge subunit [Escherichia phage FEC14]	Baseplate_J super family	cl01294
**127**	92433-93287	putative baseplate wedge subunit gp7 [Enterobacter phage phiEM4]		
**128**	93290-94480	putative tail protein [Salmonella phage Mutine]		
**129**	94527-97427	tail fibers protein [phage FEC14]		
**130**	97531-99657	tailspike protein [Salmonella phage vB_SalM_SJ3]	PhageP22-tail	pfam09251
**131**	99755-102028	hypothetical protein AML69_20060 [Escherichia coli]	Glyco_tranf_2_3 super family	cl26112
**132**	102076-104379	tail fiber protein [Escherichia phage ST31]		
**133**	104474-109312	virulence-associated VriC protein [Salmonella phage vB_SalM_PM10]	DUF4815 super family	cl24594
**134**	109365-109613	hypothetical protein STP07_201 [Salmonella phage STP07]		
**135**	109597-109938	putative capsid protein [Serratia phage phiMAM1]		
**136**	109925-110677	neck protein 2 [Salmonella phage Sh19]	13 super family	cl14347
**137**	110706-110918	hypothetical protein BSP101_0088 [Salmonella phage BSP101]		
**138**	110980-112322	neck protein [Salmonella phage Mutine]	T4_neck-protein super family	cl27828
**139**	111624-112322	putative tail sheath stabilizer [Salmonella phage BSP101]	T4-gp15_tss super family	cl14348
**140**	112325-113005	terminase DNA packaging enzyme small subunit [Salmonella phage GG32]	DNA_Packaging super family	cl27835
**141**	112986-115196	terminase DNA packaging enzyme large subunit [Salmonella phage S8]	Hint	cd00081
**142**	115249-117144	tail sheath protein [Escherichia phage FEC14]	Phage_sheath_1 super family	cl01389
**143**	117204-117389	putative homing endonuclease SegD [Salmonella phage vB_SalM_SJ3]	GIY-YIG_SF super family	cl15257
**144**	117465-117662	putative endonuclease [Escherichia phage PhaxI]		
**145**	117693-118226	Gp19 tail tube protein [Salmonella virus ViI]	19 super family	cl28641
**146**	118294-119976	portal protein [Salmonella phage BSP101]	20	PHA02531
**147**	120018-120188	hypothetical protein DET7_9 [Salmonella phage Det7]		
**148**	120200-120514	putative prohead core protein [Klebsiella phage May]		

**149**	120525-121190	prohead protease [Escherichia phage PhaxI]	Peptidase_S77 super family	cl11614
**150**	121237-122076	prohead core protein [Salmonella phage Mutine]	22 super family	cl20173
**151**	122168-123490	major capsid protein [Salmonella phage BSP101]	Gp23 super family	cl22495
**152**	123574-124221	GIY-YIG homing endonuclease	GIY-YIG_SF	cl15257
**153**	124282-124722	hypothetical protein DET7_18 [Salmonella phage Det7]		
**154**	124731-124961	hypothetical protein [Salmonella phage vB_SalM_PM10]		
**155**	125071-125352	hypothetical protein DET7_20 [Salmonella phage Det7]		
**156**	125412-125732	aspartate kinase [Epulopiscium sp. Nuni2H_MBin001]		
**157**	125693-126160	putative GTP-binding protein [Serratia phage vB_Sru_IME250]	DUF2493 super family	cl15723
**158**	126111-126365	hypothetical protein CPT_Mutine_080 [Salmonella phage Mutine]		
**159**	126396-127124	putative membrane protein [Shigella phage Sf14]		
**160**	127125-127778	putative conserved lipoprotein [Salmonella phage Marshall]		
**161**	127809-128306	tail completion and sheath stabilizer [Escherichia phage PhaxI]	3 super family	cl24094
**162**	128345-128800	DNA repair/recombination protein UvsY [Salmonella phage vB_SalM_SJ3]	UvsY super family	cl12619
**163**	128800-129546	PD-(D/E)XK nuclease [Salmonella phage Marshall]	Cas4_I super family	cl00641
**164**	129576-131075	DNA helicase [Salmonella phage GG32]	HsdR super family	cl28233
**165**	131081-131446	clamp loader subunit [Salmonella phage Det7]	Vault super family	cl27548
**166**	131785-132453	gp45 sliding clamp holder [Salmonella phage Sh19]	gp45-slide_C super family	cl27865
**167**	132531-133520	clamp loader subunit, ATPase [Salmonella phage Marshall]	Rad17 super family	cl25702
**168**	133525-133947	clamp holder for DNA polymerase [Salmonella phage SP1]	Phage_clamp_A super family	cl27778
**169**	133977-134441	RegA translational repressor protein [Shigella phage Ag3]	regA	PHA02543
**170**	134458-135321	MazG-like domain [Salmonella phage Det7]	NTP-PPase super family	cl16941
**171**	135392-136597	hypothetical protein [Escherichia phage PhaxI]		
**172**	137098-139113	DNA-directed RNA polymerase [Raoultella phage Ro1]	DUF2135 super family	cl19870
**173**	139152-139523	hypothetical protein SP063_00812 [Salmonella phage FSL SP-063]		

**174**	139583-139957	hypothetical protein [Salmonella phage vB_SalM_PM10]	DUF3268 super family	cl13172
**175**	139966-140838	hypothetical protein [Salmonella phage vB_SalM_PM10]	Phage_gp49_66 super family	cl10351
**176**	140988-141164	hypothetical protein Marshall_86 [Salmonella phage Marshall]		
**177**	141157-143373	von Willebrand factor type A domain protein [Dickeya phage RC-2014]	vWFA super family	cl00057
**178**	143417-143728	acyl carrier protein [Salmonella phage FSL SP-063]	PP-binding super family	cl09936
**179**	143860-144099	hypothetical protein CPT_Mutine_105 [Salmonella phage Mutine]		
**180**	144159-144614	deoxyribonuclease [Comamonadaceae bacterium NML79-0751]	Pyr_excise super family	cl19573
**181**	144627-144905	hypothetical protein [Salmonella phage vB_SalM_PM10]		
**182**	144943-145392	PLxRFG domain-containing protein [Ottowia thiooxydans]		
**183**	145477-146166	i-spanin [Salmonella phage Marshall]		
**184**	146163-146483	o-spanin [Salmonella phage Marshall]		
**185**	146533-147324	hypothetical protein [Salmonella phage GG32]		
**186**	147381-148049	ImpD [Salmonella phage Sh19]		
**187**	148051-148338	hypothetical protein S8_006 [Salmonella phage S8]		
**188**	148-328-148648	superinfection exclusion protein [Salmonella phage Sh19]	PHA01516 super family	cl10305
**189**	148645-148854	unnamed protein product [Salmonella phage SFP10]		
**190**	148854-149645	SPFH domain band 7 family protein [Erwinia phage phiEa2809]	SPFH_like super family	cl19107
**191**	149729-150007	HU_IHF DNA binding domain protein [Salmonella phage Det7]	HU	cd13831
**192**	150143-151864	DNA helicase [Salmonella phage vB_SalM_PM10]	DinG super family	cl26946
**193**	151861-152628	hypothetical protein [Salmonella phage SP1]		
**194**	152671-153198	ribonuclease HI [Salmonella phage vB_SalM_SJ2]	RNase_HI_prokaryote_like family	cd09278
**195**	153209-153931	putative homing endonuclease [Salmonella phage Sh19]		
**196**	153931-154725	sigma factor for late transcription [Salmonella phage Sh19]	55 super family	cl14345
**197**	154712-155827	recombination protein subunit [Salmonella phage vB_SalM_SJ3]	47 super family	cl26377

Table S3. Comparison of phages against the LPST94 genome.

Sl. No	Phage	Isolated place	Accession	Length (bp)	BlastN score	E value	Query coverage	Identity	Family	Ref.
1	*Salmonella* phage PhiSH19	UK	JN126049	157785	2.28E+05	0	88%	98%	*Ackermannviridae*	(Hooton et al., 2011)
2	*Salmonella* phage STP07	Korea	KY000003	160342	2.34E+05	0	89%	99%	*Ackermannviridae*	Unpublished
3	*Escherichia* phage PhaxI	Iran	JN673056	156628	2.25E+05	0	89%	97%	*Ackermannviridae*	(Salehe Sabouri et al., 2013)
4	*Salmonella* phage SFP10	Korea	HQ259103	157950	2.34E+05	0	89%	98%	*Ackermannviridae*	(Minjung et al., 2012)
5	*Escherichia* virus CBA120	USA	JN593240	157304	2.30E+05	0	89%	97%	*Ackermannviridae*	(Kutter et al., 2011)
6	*Escherichia* phage FEC14	China	MG383452	158639	2.24E+05	0	87%	97%	*Ackermannviridae*	Unpublished

Table S4. Antibiotics resistance profiles of the clinical isolates of *Salmonella*.

Strains	Serovars	CTT	AMP	CZO	CAZ	FEP	GEN	AMK	TOB	CRO	ATM	CIP	SXT	NIT
LST10	*S.* Typhimurium	R	R	R	S	S	R	R	R	S	S	R	S	R
LST11	*S.* Typhimurium	R	R	R	S	S	R	R	R	S	S	R	R	R
LST12	*S.* Typhimurium	S	S	R	S	S	S	S	S	S	S	S	S	R
LST13	*S.* Typhimurium	S	R	S	S	S	S	S	S	S	S	S	R	S
LST14	*S.* Typhimurium	S	S	R	R	R	S	S	R	R	S	S	R	S
LST15	*S.* Typhimurium	S	R	S	S	S	S	S	S	S	S	S	S	R
LST16	*S.* Typhimurium	S	R	S	S	S	R	S	R	S	S	S	S	S
LST17	*S.* Typhimurium	S	R	R	R	R	S	S	R	R	R	S	S	S
LST18	*S.* Typhimurium	R	R	R	S	S	R	R	R	S	S	R	R	R
LST19	*S.* Typhimurium	R	R	R	S	S	R	R	R	R	S	R	S	R
LSE6	*S.* Enteritidis	R	R	R	S	S	R	R	R	S	S	R	R	R
LSE7	*S.* Enteritidis	S	R	S	S	S	S	S	S	S	S	S	S	R
LSE8	*S.* Enteritidis	S	R	S	S	S	S	S	S	S	S	S	S	R
LSE9	*S.* Enteritidis	R	R	R	S	S	R	R	R	S	S	S	R	R
LSE10	*S.* Enteritidis	S	R	R	S	S	S	S	S	S	S	S	S	R
LSE11	*S.* Enteritidis	S	R	R	S	S	S	S	S	S	S	S	S	R
LSE12	*S.* Enteritidis	R	S	S	S	S	S	S	S	S	S	S	S	R
LSE15	*S.* Enteritidis	S	R	R	S	S	R	R	R	S	S	S	S	R

Abbreviations: *S, Salmonella*; S, susceptible; R, resistant; CTT, cefotetan; AMP, ampicillin; CZO, cefazolin; CAZ, ceftazidime; FEP, cefepime; GEN, gentamicin; AMK, amikacin; TOB, tobramycin; CRO, ceftriaxone, ATM, aztreonam; CIP, ciprofloxacin; SXT, paediatric compound sulfamethoxazole tablets; NIT, nitrofurantoin.
